# Supplementary material for: In vivo self-renewal and expansion of quiescent stem cells from a non-human primate
Source: Nat Commun. 2025 Jun 24;16:5370. doi: 10.1038/s41467-025-58897-x (PMC12187938; doi:10.1038/s41467-025-58897-x)
Supplement: Supplementary file 2 — Description of Addtional Supplementary Files [file 41467_2025_58897_MOESM2_ESM.pdf]

Description of Additional Supplementary Files

Supplementary Data 1: Antibody clones and other tools for mouse lemur. List of antibody clones and their ability to cross react with mouse lemur protein. Included are multiple sequence alignments of the amino acid sequence of the epitope targeted by the indicated antibodies, for those antibodies for which the epitope is known.

Supplementary Data 2: Gene enrichment analysis for mouse lemur MuSCs and FAPs List of top enriched genes in SmartSeq2 for FACS purified $NCAM1^{+}/THY1^{-}$ (MuSCs) and $NCAM1^{-}$ /$THY1^{+}$ (FAPs) cells.

Supplementary Data 3: Expression levels of cell-type marker genes. Top significantly enriched genes for each cell type. The table has tabs listing genes that are higher in human & lemur compared to mouse, or human & mouse compared to lemur, or lemur & mouse compared to human, or that are higher in one of the individual species.

Supplementary Data 4: Differentially expressed genes in MuSCs, FAPs, and myofibers. Top significantly enriched genes for MuSCs, FAPs, and myofibers. Two-tailed Wilcoxon rank-sum tests comparing expression independently for each gene and each homologous cell type with $p < 1e-5$.

Supplementary Data 5: Gene enrichment analysis for macaque-specific cell types. List of top enriched genes in 10X single cell RNAseq data for the major cell types identified in macaque muscle, including two cell types unique to macaque: a subtype of capillary endothelial cells and a subtype of pericyte.

Supplementary Data 6: Four-species gene expression analyses. Gene expression levels for all conserved genes across the identified cell types across the four species. The first tab lists the number of cells used for the analysis for each cell type in each species. Subsequent tabs show the mean expression or the percent positive cells for each of the four species.

Supplementary Data 7: Disease gene expression levels across single cells. Genes are categorized by the main cell type in which we hypothesized the genetic defect to manifest, based on functional studies, gene expression reports, and phenotypic descriptions. For each gene, the disease name is listed as annotated on MIM. Only one disease is listed when a gene is linked to multiple. Functional categorization of gene function was based on Gene Cards. Final columns list references for the gene mutation and the mouse model. Disease gene expression levels. For each gene which an ortholog had been annotated in the human, mouse lemur, and mouse genomes, the expression levels were calculated.

Supplementary Data 8: Gene expression levels for primate-specific genes in indicated skeletal muscle cell types.

Supplementary Movie 1: Video recording of contracting mouse lemur myotubes.
