# Supplementary material for: In vivo self-renewal and expansion of quiescent stem cells from a non-human primate
Source: Nat Commun. 2025 Jun 24;16:5370. doi: 10.1038/s41467-025-58897-x (PMC12187938; doi:10.1038/s41467-025-58897-x)
Supplement: Supplementary file 12 — Reporting Summary [file 41467_2025_58897_MOESM12_ESM.pdf]

Corresponding author(s): Antoine de Morree
Thomas Rando

Last updated by author(s): Dec 14, 2024

Reporting Summary

Nature Portfolio wishes to improve the reproducibility of the work that we publish. This form provides structure for consistency and transparency in reporting. For further information on Nature Portfolio policies, see our Editorial Policies and the Editorial Policy Checklist.

Statistics

For all statistical analyses, confirm that the following items are present in the figure legend, table legend, main text, or Methods section.

n/a	Confirmed	
☐	☒	The exact sample size (*n*) for each experimental group/condition, given as a discrete number and unit of measurement
☐	☒	A statement on whether measurements were taken from distinct samples or whether the same sample was measured repeatedly
☐	☒	The statistical test(s) used AND whether they are one- or two-sided<br>*Only common tests should be described solely by name; describe more complex techniques in the Methods section.*
☒	☐	A description of all covariates tested
☐	☒	A description of any assumptions or corrections, such as tests of normality and adjustment for multiple comparisons
☐	☒	A full description of the statistical parameters including central tendency (e.g. means) or other basic estimates (e.g. regression coefficient) AND variation (e.g. standard deviation) or associated estimates of uncertainty (e.g. confidence intervals)
☐	☒	For null hypothesis testing, the test statistic (e.g. *F*, *t*, *r*) with confidence intervals, effect sizes, degrees of freedom and *P* value noted<br>*Give P values as exact values whenever suitable.*
☒	☐	For Bayesian analysis, information on the choice of priors and Markov chain Monte Carlo settings
☒	☐	For hierarchical and complex designs, identification of the appropriate level for tests and full reporting of outcomes
☒	☐	Estimates of effect sizes (e.g. Cohen's *d*, Pearson's *r*), indicating how they were calculated

Our web collection on statistics for biologists contains articles on many of the points above.

Software and code

Policy information about availability of computer code

Data collection	FACS and flow cytometry: FACSDIVA 8+ (BD Bioscience)<br>Fluorescent microscope images: CellReporterXpress (Molecular devices), Volocity 5+ (PerkinElmer), ZEN 2.3 (Carl Zeiss), BX63 (Olympus)<br>RT-qPCR: LightCycler 480 1.5.0 SP3 (Roche)<br>BLI imaging: IVIS Living Image Software, version 4.0 (Caliper Life Sciences, Perkin Elmer)<br>Microfludic PCR: Biomark (Fluidigm)<br>Single cell western: Milo (ProteinSimple)
Data analysis	Image analysis: Volocity 5+ (PerkinElmer), Image J 1.53t (National Institutes of Health), CellReporterXpress (Molecular devices), Huygens Essential 16.10.1p4 (Scientific Volume Imaging B. V.), ImageJ Fiji<br>Flow cytometry: FlowJo v10.8.1 (BD Biosciences)<br>BLI imaging: IVIS Lumina Series 4.8.0 (PerkinElmer)<br>CyTOF data: Cell Engine (Cellcarta)<br>Statistics: Excel (Microsoft), Prism 10 (Graphpad)

For manuscripts utilizing custom algorithms or software that are central to the research but not yet described in published literature, software must be made available to editors and reviewers. We strongly encourage code deposition in a community repository (e.g. GitHub). See the Nature Portfolio guidelines for submitting code & software for further information.

Data

Policy information about availability of data

All manuscripts must include a data availability statement. This statement should provide the following information, where applicable:

- Accession codes, unique identifiers, or web links for publicly available datasets
- A description of any restrictions on data availability
- For clinical datasets or third party data, please ensure that the statement adheres to our policy

Tabula Microcebus mouse lemur scRNAseq gene expression counts/UMI tables, and cellular metadata used in this study are available on "figshare [https://figshare.com/projects/Tabula_Microcebus/112227]". For the cross-species comparison, human data were from the 10x data of the "Tabula Sapiens for the muscle [https://figshare.com/projects/Tabula_Sapiens/100973]". Mouse data were from 10x data of the "Tabula Muris Senis [https://figshare.com/articles/dataset/Processed_files_to_use_with_scanpy_/8273102/2]". Crab-eating macaque data were from the 10x data of the cynomolgus monkey cell atlas, available on "Zenodo [https://zenodo.org/records/5881495#.ZERMCnbMKUk]".
All other data supporting the findings of this study are available within the article and its supplementary files. Source data are provided with this paper. Any additional requests for information can be directed to, and will be fulfilled by, the corresponding authors.

Research involving human participants, their data, or biological material

Policy information about studies with human participants or human data. See also policy information about sex, gender (identity/presentation), and sexual orientation and race, ethnicity and racism.

Reporting on sex and gender	Sex of all donors is reported in the methods section
Reporting on race, ethnicity, or other socially relevant groupings	*Please specify the socially constructed or socially relevant categorization variable(s) used in your manuscript and explain why they were used. Please note that such variables should not be used as proxies for other socially constructed/relevant variables (for example, race or ethnicity should not be used as a proxy for socioeconomic status). Provide clear definitions of the relevant terms used, how they were provided (by the participants/respondents, the researchers, or third parties), and the method(s) used to classify people into the different categories (e.g. self-report, census or administrative data, social media data, etc.) Please provide details about how you controlled for confounding variables in your analyses.*
Population characteristics	*Describe the covariate-relevant population characteristics of the human research participants (e.g. age, genotypic information, past and current diagnosis and treatment categories). If you filled out the behavioural & social sciences study design questions and have nothing to add here, write "See above."*
Recruitment	The muscle biopsy samples were donated via Donor Network West (DNW).
Ethics oversight	Donor Network West internal ethics committee; Institutional Review Board at Stanford University.

Note that full information on the approval of the study protocol must also be provided in the manuscript.

Field-specific reporting

Please select the one below that is the best fit for your research. If you are not sure, read the appropriate sections before making your selection.

☒ Life sciences ☐ Behavioural & social sciences ☐ Ecological, evolutionary & environmental sciences

For a reference copy of the document with all sections, see nature.com/documents/nr-reporting-summary-flat.pdf

Life sciences study design

All studies must disclose on these points even when the disclosure is negative.

Sample size	No sample-size calculation was performed. Sample sizes were chosen based on the types of experiment, availability of animals, and standard practice when using individual animals as biological replicates (general range n=3-4). Sample size was informed by availability (mouse lemur tissues were harvested opportunistically).
Data exclusions	No data were excluded from the analyses.
Replication	All experiments were replicated across multiple individuals. Reproducibility was ensured by the use of complementary assays. Experiments were reproducible across different days and investigators.
Randomization	No randomization was used. Samples were split into control and treated groups.
Blinding	Investigators were blinded during data analysis. Clear cutoffs and automated analyses were used to minimize the effects of investigator bias.

Reporting for specific materials, systems and methods

We require information from authors about some types of materials, experimental systems and methods used in many studies. Here, indicate whether each material, system or method listed is relevant to your study. If you are not sure if a list item applies to your research, read the appropriate section before selecting a response.

Materials & experimental systems

n/a	Involved in the study
☐	☒ Antibodies
☒	☐ Eukaryotic cell lines
☒	☐ Palaeontology and archaeology
☐	☒ Animals and other organisms
☒	☐ Clinical data
☒	☐ Dual use research of concern
☒	☐ Plants

Methods

n/a	Involved in the study
☒	☐ ChIP-seq
☐	☒ Flow cytometry
☒	☐ MRI-based neuroimaging

Antibodies

Antibodies used

Antibodies are listed in the manuscript in Supplementary Data 1.
FITC-anti-CD31 (BioLegend; #102506, clone MEC13.3)
FITC-anti-CD45 (BioLegend; #103108, clone 30-F11)
Pacific blue-anti-Sca1 (BioLegend; #108120, clone D7)
PE/Cy7-anti-VCAM (BioLegend; #105720, clone 429)
APC-anti-human CD31 (BioLegend; #303116, clone WM59)
FITC-anti-human CD45 (Invitrogen; #MHCD4501, clone HI30)
PE-anti-human CD90 (BioLegend; #328110, clone 5E10)
PE/Cy7-anti-human CD82 (BioLegend; #342110, clone ASL-24)
CD29 (ITGB1) mouse TS2/16 BioLegend
CD34 mouse 581 BioLegend
CD82 mouse ASL-24 BioLegend
CFD rabbit PA5-79034 Sigma
CXCR4 mouse 12G5 BioLegend
GLIS3 rabbit HPA056426 Sigma
HNRNPA1 rabbit HPA007185 Sigma
MYH2 mouse 2F7 DSHB
MyoD mouse 5.8A BD Biosciences
MyoG mouse F5D BD Biosciences
NCAM1 mouse MEM-188 BioLegend
OAZ1 rabbit NBP1-88925 Novus
PAX7 mouse PAX7-c DSHB
PDGFRA rabbit ab134123 Abcam
PLIN1 rabbit D1D8 Cell Signaling
RUNX2 rabbit HPA022040 Sigma
SAT1 rabbit NB110-41622 Novus
SGCA rabbit HPA007537 Sigma
SMA rabbit D4K9N Cell Signaling
SP7rabbit HPA063202 Sigma
Spermidine rabbit NB100-1847 Novus
SRM rabbit ab241508 Abcam
TUBB rabbit ab6046 Abcam
anti-luciferase #L0159 Sigma-Aldrich
anti-GFP A11122 Invitrogen
anti-rabbit-biotin A16039 Invitrogen
anti-rabbit-647 A31573 Invitrogen
anti-rabbit-488, A21206 Invitrogen
anti-mouse-488 A21202 Invitrogen
anti-mouse-647 A-31571 Invitrogen

Validation

Species reactivity is listed in the manuscript Supllementary Data 1
FITC-anti-CD31 (MEC13.3), FITC-anti-CD45 (30-F11), and Pacific blue-anti-Sca1 (D7) were validated on mouse splenocytes by manufacturer and have been characterized in its use for FACS-isolating mouse muscle stem cells in Liu et al., 2013 Nature Protocols.
PE/Cy7-anti-VCAM (429) was validated on mouse bone marrow cells by manufacturer and have been characterized in its use for FACS-isolating mouse muscle stem cells in Liu et al., 2013 Nature Protocols.
APC-anti-human CD31 (WM59) was validated on human peripheral blood lymphocytes, monocytes, and granulocytes by manufacturer, and have been characterized in its use for FACS-isolating human muscle stem cells in Charville et al., 2015 Stem Cell Reports.
FITC-anti-human CD45 (HI30) was validated in rabbit amniotic cells, spiked cells, and human bone marrow samples by external data collected by manufacturer, and have been characterized in its use for FACS-isolating human muscle stem cells in Charville et al., 2015

Stem Cell Reports.
PE-anti-human CD90 (5E10) was validated on human erythroleukemic cell line by manufacturers, and have been characterized in its use for FACS-isolating human muscle stem cells in Charville et al., 2015 Stem Cell Reports.
PE/Cy7-anti-human CD82 (ASL-24) was validated on human peripheral blood lymphocytes by manufacturer, and have been characterized in its use for FACS-isolating human muscle stem cells in Charville et al., 2015 Stem Cell Reports.
PE/Cy7-anti-human-NCAM1 (MEM-188) Each lot is quality control tested by immuno-fluorescent staining with flow cytometric analysis by manufacturer
Pax7 (Pax7-c) was validated in various species including human and mouse by manufacturer and extensive use in other studies.
Spermidine synthase (ab241508) was validated in various cell lines by manufacturer and kang et al 2024 Nature Metabolism.
Sat1 (NB110-41622) was validated in mouse seminal vesical and prostate tissue by manufacturer, and extensive use in other studies.
OAZ1 (NBP1-88925) was vaildated in human skeletal muscle by manufacturer.
Spermidine (ab7318) was validated in rat lung tissue by manufacturer and other studies.
CFD (PA5-79034) was validated in mouse small intestine tissue by manufacturer
CXCR4 (12G5) was validated in knockout cells by manufacturer
GLIS3 (HPA056426) was validated in human galbladder by manufacturer
HNRNPA1 (HPA007185) was validated in U2OS cells by manufacturer
MYH2 (2F7) was validated in mouse muscle by manufacturer
MyoD (5.8A) validated on MyoD knockout cells in de Morree et al, 2017, PNAS.
MyoG (F5D) was validated in mouse c2c12 cells by manufacturer
PDGFRA (ab134123) was validated in human colon tissue by manufacturer
PLIN1 (D1D8) was validated in mouse brown fat by manufacturer
RUNX2 (HPA022040) was validated in human lymph node by manufacturer
SGCA (HPA007537) was validated in human heart muscle by manufacturer
SMA (D4K9N) was validated in mouse small intestine by manufacturer
SRM (ab241508) was validated in HEK293T cells by manufacturer
SP7/Osteryx (HPA063202) was validated in human testis by manufacturer
TUBB (ab6046) was validated in immunoprecipitation experiments by manufactorer

Animals and other research organisms

Policy information about studies involving animals; ARRIVE guidelines recommended for reporting animal research, and Sex and Gender in Research

Laboratory animals	Microcebus murinus; Macaca fascicularis; Mus musculs;<br>Old mice used in this study were obtained from CHarles Rives Laboratories through the National Institute on Aging. NSG mice (#005557) were purchased from The Jackson Laboratory.
Wild animals	This study does not involve wild animals.
Reporting on sex	Males and females were used
Field-collected samples	This study does not involve samples collected from the field.
Ethics oversight	All animal studies were approved by local ethics committees. Stanford University Administrative Panel on Laboratory Animal Care; Administrative Panel on Laboratory Animal Care of the VA Palo Alto Health Care System; Animal Welfare Inspectorate Denmark; Animal Welfare Board at Aarhus University.

Note that full information on the approval of the study protocol must also be provided in the manuscript.

Plants

Seed stocks	*Report on the source of all seed stocks or other plant material used. If applicable, state the seed stock centre and catalogue number. If plant specimens were collected from the field, describe the collection location, date and sampling procedures.*
Novel plant genotypes	*Describe the methods by which all novel plant genotypes were produced. This includes those generated by transgenic approaches, gene editing, chemical/radiation-based mutagenesis and hybridization. For transgenic lines, describe the transformation method, the number of independent lines analyzed and the generation upon which experiments were performed. For gene-edited lines, describe the editor used, the endogenous sequence targeted for editing, the targeting guide RNA sequence (if applicable) and how the editor was applied.*
Authentication	*Describe any authentication procedures for each seed stock used or novel genotype generated. Describe any experiments used to assess the effect of a mutation and, where applicable, how potential secondary effects (e.g. second site T-DNA insertions, mosiacism, off-target gene editing) were examined.*

Flow Cytometry

Plots

Confirm that:

☒ The axis labels state the marker and fluorochrome used (e.g. CD4-FITC).

☒ The axis scales are clearly visible. Include numbers along axes only for bottom left plot of group (a 'group' is an analysis of identical markers).

☒ All plots are contour plots with outliers or pseudocolor plots.

☒ A numerical value for number of cells or percentage (with statistics) is provided.

Methodology

Sample preparation	Skeletal muscles were harvested postmortem by an experienced veterinarian. Tissues were mechanically dissociated and digested with Collagenase II. A second digestion was performed with Collagenase II and Dispase. The resulting cell suspensions were pulled through 25- gauge needles and pushed through 100 µm and 40 µm filters. The resulting single cell suspensions were stained with antibodies for 15 min at 4°C with head-over-head rotation. Cells were washed, filtered, and analyzed on calibrated BD-FACS Aria II or BD FACSAria III flow cytometers equipped with 488-nm, 633-nm and 405-nm lasers.
Instrument	BD Aria III
Software	FACSDIVA 8+ (BD Biosciences) and FlowJo
Cell population abundance	MuSCs (~0.5-1%) and FAPs (~1-2%) purity was assessed by re-sort or by antibody staining of the sorted cells, as detailed in the manuscript.
Gating strategy	Mouse lemur: Gating for CD90 or CD56<br>Mouse: Negative selection for CD31 and CD45. Positive selection for Ly6/A (Sca1) or VCAM1.<br>Human: Negative selection for CD31 and CD45. Positive selection for CD90 or CD82

☒ Tick this box to confirm that a figure exemplifying the gating strategy is provided in the Supplementary Information.
